# Supplementary material for: Potential Therapeutic Use of Aptamers against HAT1 in Lung Cancer
Source: Cancers (Basel). 2022 Dec 30;15(1):227. doi: 10.3390/cancers15010227 (PMC9818519; doi:10.3390/cancers15010227)
Supplement: Supplementary file 1 [file cancers-15-00227-s001.zip › cancers-2027091-supplementary.pdf]

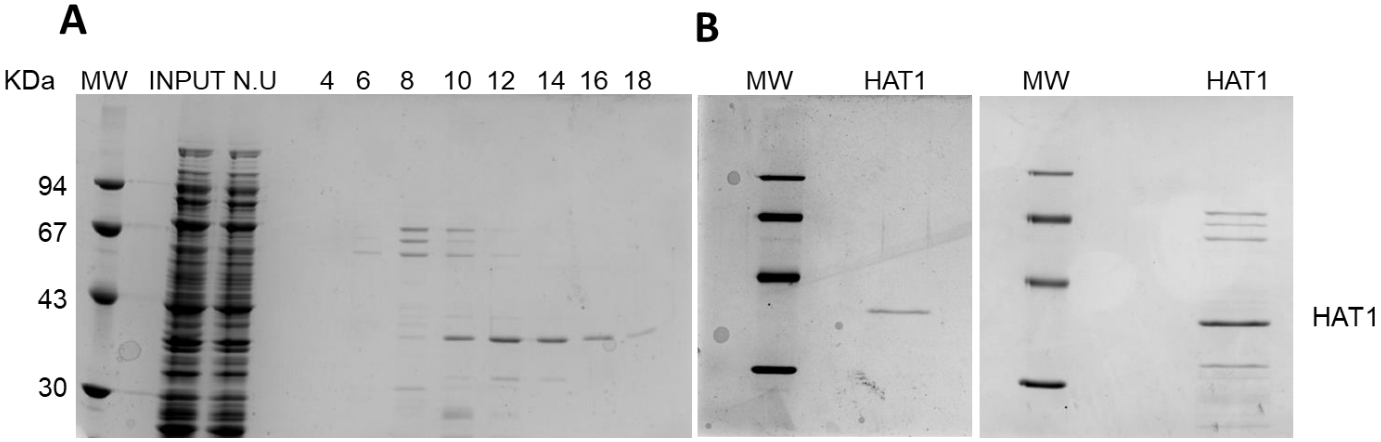

Figure S1. Purification of recombinant His-HAT1 protein. (A) SDS-PAGE 12% polyacrylamide gels stained with Coomassie blue showing the fractions where eluted His-HAT1 (B) Fractions containing His-HAT1 were pooled, resolved in SDS-PAGE and stained with Coomassie blue to determine the purity and quantify the amount of purified protein. The recombinant protein has a purity of 68-85% and an expected molecular weight of 37.7 kDa. MW: Molecular weight marker. N.U. non-bound.

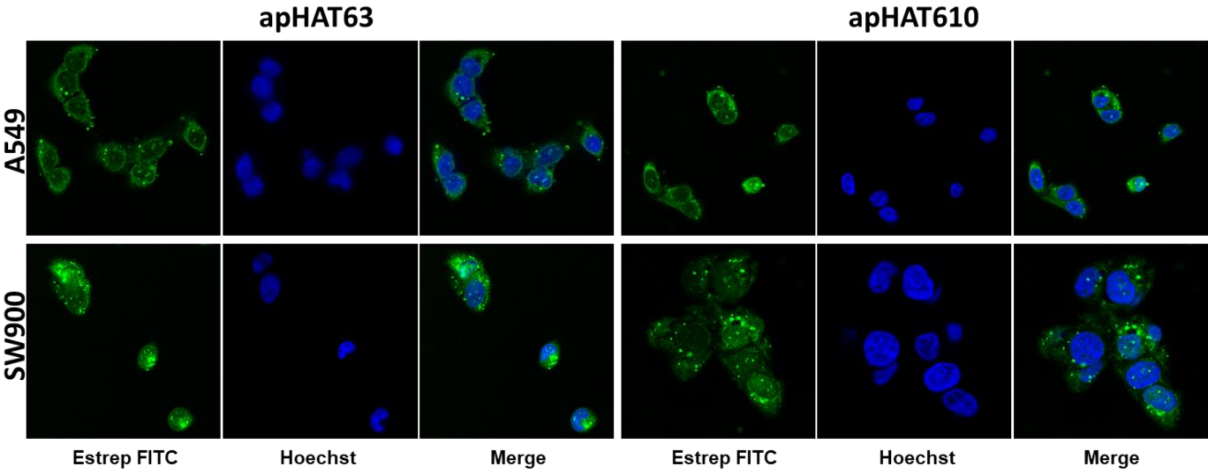

Figure S2. Cellular uptake and subcellular localization of the aptamers. The entry of the aptamers into the cell was confirmed after transfection of the cells A549 and SW900 with biotin-conjugated aptamers. Twenty-four hours post transfection, cells were fixed with absolute methanol and incubated with streptavidin-HRP 1/200. Finally, the cells were mounted on glass slides using glycerol-buffer containing p-phenyl-enediamine and 30 µmol/l bis-benzamide (Hoechst 33342) for nuclear staining. Confocal microscopy images corresponding to the staining of nuclei with Hoechst (blue) and aptamers (green). Objective 60X. The aptamers are located in the cytoplasm and the nucleus in both cell lines.

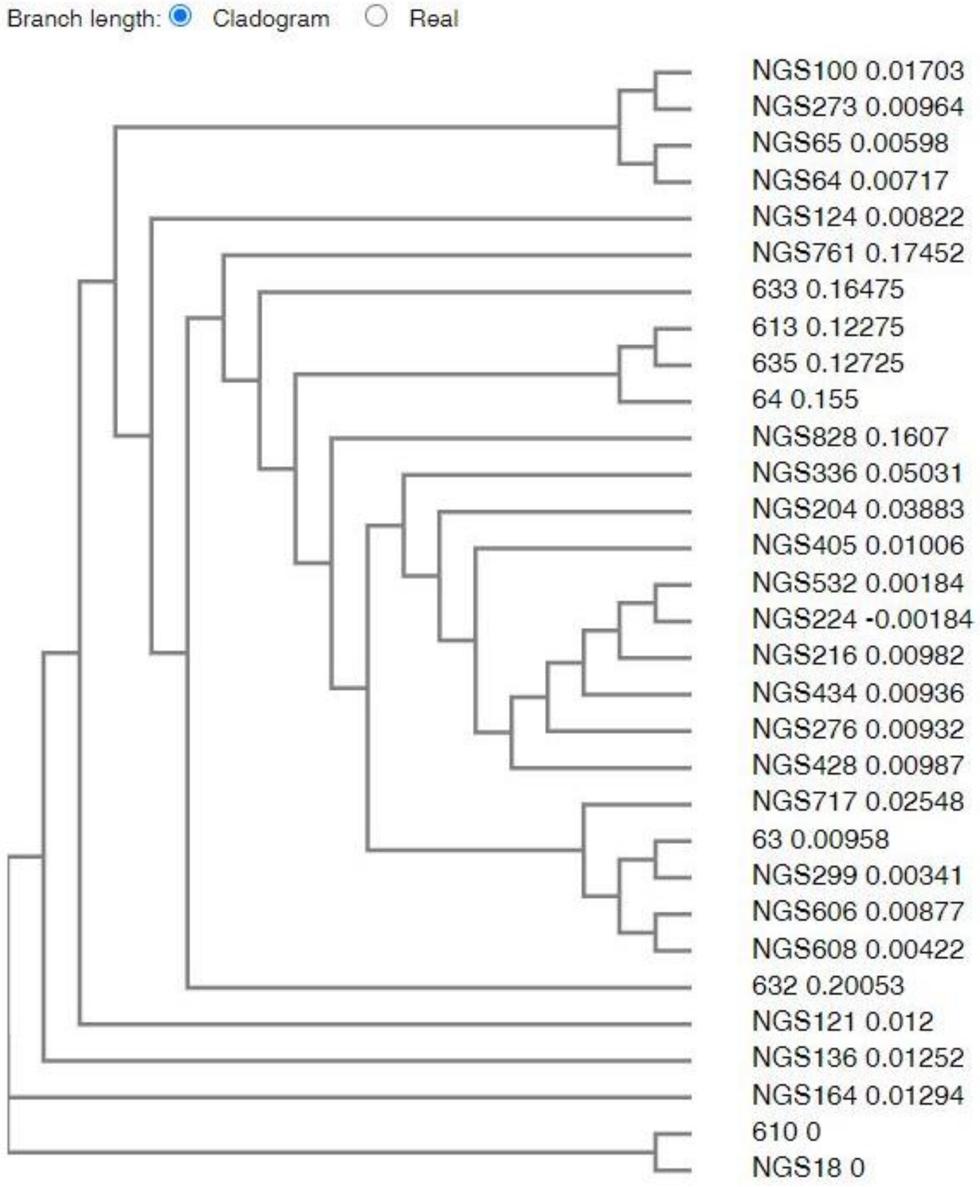

Figure S3. Phylogenetic dendrogram. A Neighbour-joining tree without distance corrections was generated using Clustal tools for multiple sequences alignment (EMBL-EBI Services, https://www.ebi.ac.uk/Tools/msa/clustalo/). The twenty-four sequences more represented in the NGS analysis and the seven sequences from cloning were compared.

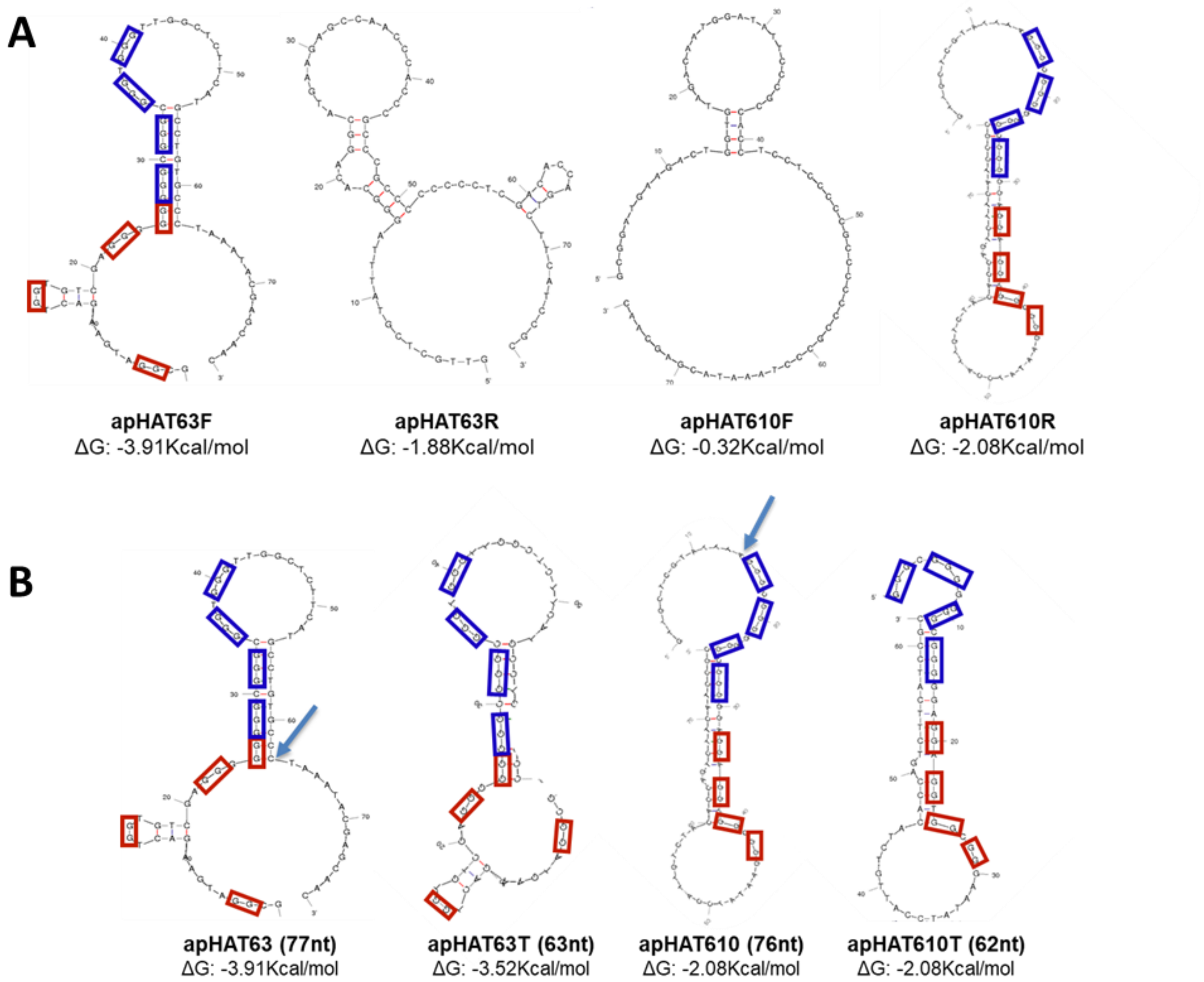

Figure S4. Secondary and G-quadruplex structures. Possible prediction of secondary structures and G-quadruplexes of selected aptamers by bioinformatic analysis of sequences with mFold and QGRS Mapper programs. Red and blue boxes indicate the G-quadruplex groups in the (A) apHAT63 and apHAT610 F and R sequences, and (B) apHAT63 and apHAT610 and their truncates. Arrows in the parental aptamers indicate the sequence cleavage site.

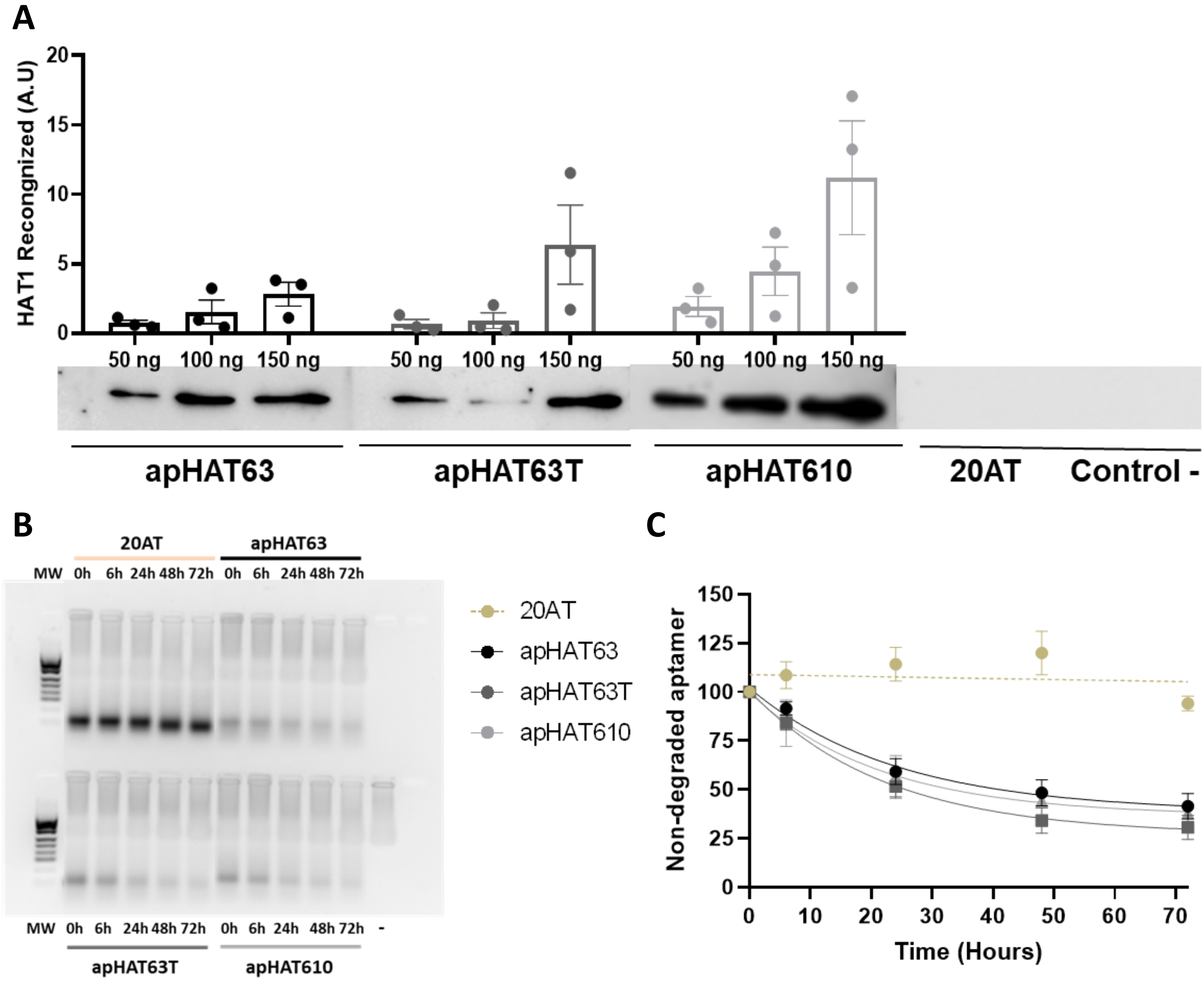

Figure S5. Affinity of the aptamers by denatured HAT1 and stability in plasma (A) Apta-western blot with increasing concentrations of recombinant HAT1 protein at 50, 100 and 150 ng (1.25, 2.5 and 3.75 pmoles) that were incubated with a 50 nM concentration of each aptamer. The graph represents the mean ± SEM of 3 independent experiments. The image corresponds to a representative experiment. (B) Stability of aptamers in human plasma. The aptamers (600 ng) were incubated in the pres-ence of human plasma, samples were obtained at 0h, 6h, 24h, 24h, 48h and 72h and loaded on a 3% agarose gel. The negative control (-) consists of buffer and human plasma. The image corre-sponds to a representative assay. (C) Quantification of the amount of undegraded aptamer. Data are expressed as percentage of undegraded aptamer with respect to time 0h. The graph repre-sents the mean ± SEM of 3 independent experiments.

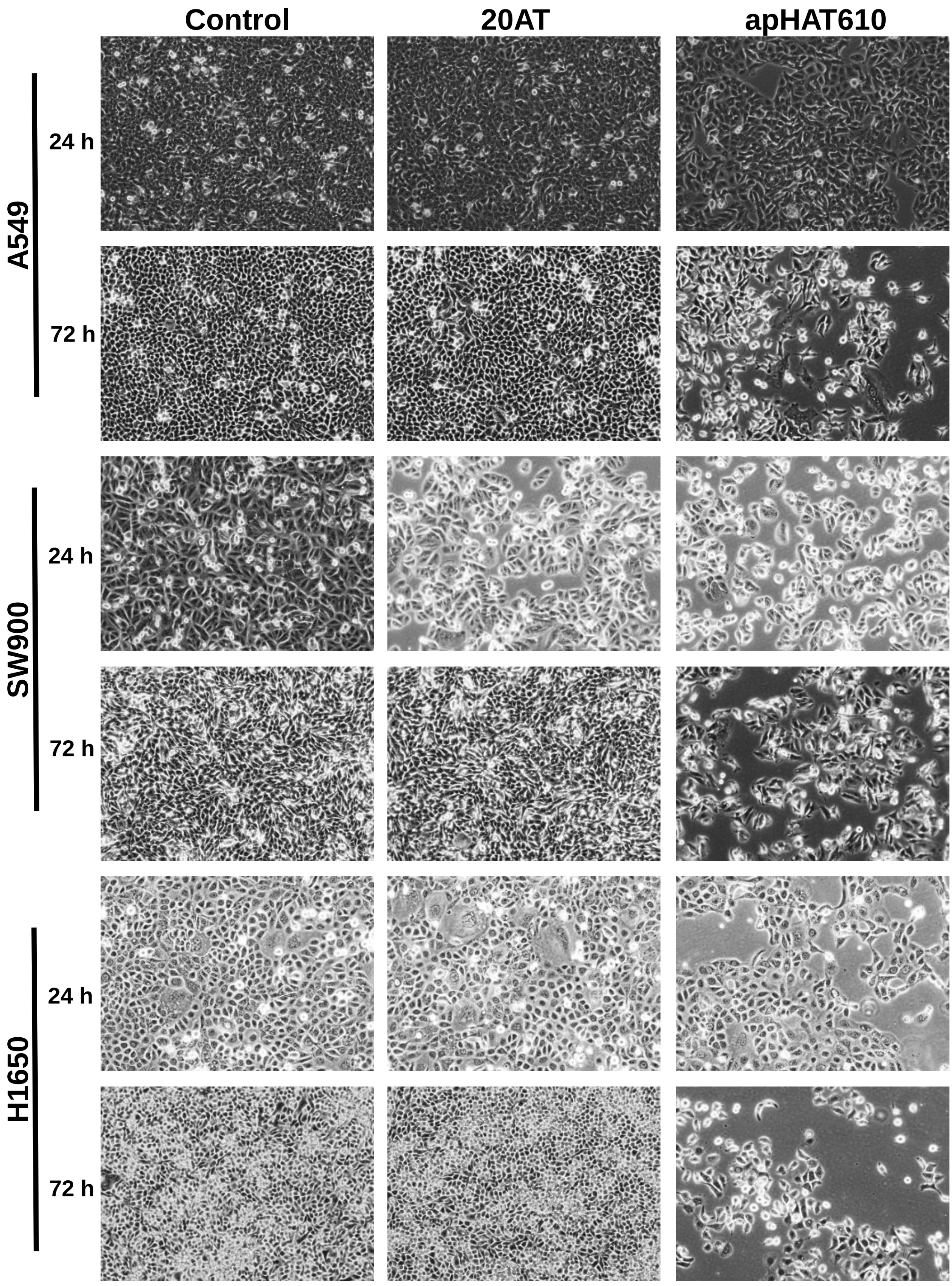

Figure S6. Effect of apHAT610 on cell growth of the 3 study lines. A549, SW900, and H1650 cells were seeded in p96 at a density of 6x103 cells/well for A549 and 104 cells/well for the other two lines. After 24 hours they were transfected with apHAT610 at a concentration of 2 times the IC50 for each line, and at the 24 and 72-hour times microscopy images were taken at 10X.

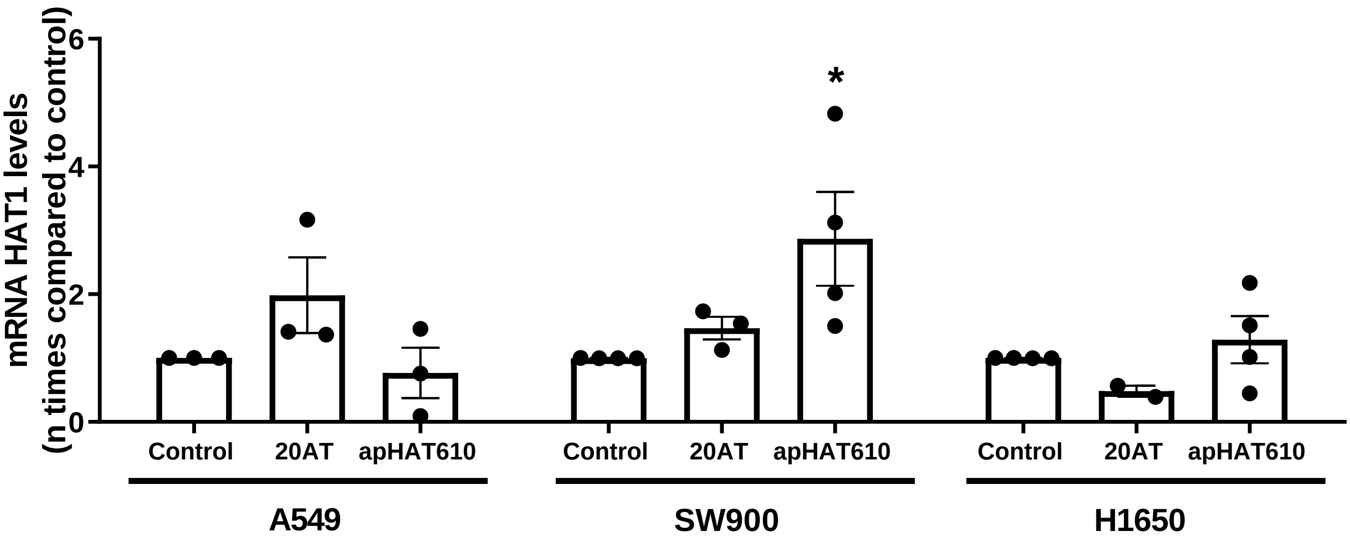

Figure S7. Effect of apHAT610 on HAT1 mRNA levels. A549, SW900, and H1650 cells were seeded on p6 at density 5 x 105 cells/well and transfected at 24 hours with apHAT610 at previously established working concentrations. After 24 hours, the cells were lifted, and their RNA was extracted. The graph shows the quantification of HAT1 messenger RNA levels by qRT-PCR. Data are expressed as 2-ΔΔΔCt and represent the mean ± SEM of 3 independent experiments (*p<0.05 relative to control).

Table S1. List of oligonucleotides used in this study

NAME	SEQUENCE
F3	GCGGATGAAGACTGGTGT
R3	GTTGCTCGTATTTAGGGC
T7	TAATACGACTCACTATAGGG
Sp6	ATTTAGGTGACACTATAGAA
5'HAT1	GGATGGAGCTACGCTCTTTG
3'HAT1	GGATGGATCTTCCGCTGTAA
5'β-actin	TGGACTTCGAGCAAGAGATG
3'β-actin	GAAGGAAGGCTGGAAGAGTG

Table S2. List of antibodies used in this study

Antibody	Company	MW(kDa)	Clonality/Reactivity	Dilution
β-actin	Sigma Aldrich, USA	45	Monoclonal/Mouse	1/10000
Cleaved PARP (Asp ^{214})	Cell Signaling, USA	89	Policlonal/Rabbit	1/1000
HAT1	Genetex, USA	50	Policlonal/Rabbit	1/5000
Acetyl-Histone H4 (Lys5, Lys8, Lys12, Lys16)	Invitrogen, USA	15	Policlonal/Rabbit	1/5000
Histone H4	Abcam, UK	11	Policlonal/Rabbit	1/1000

Table S3. List of aptamers used in this study

Aptamer	Sequence
apHAT63	GCGGATGAAGACTGGTGTCGAGGGGGGGGCGGGCGGGTGGGTTGGCT CTTCATGCCTGTGCCCTAAATACGAGCAAC
apHAT63T	GCGGATGAAGACTGGTGTCGAGGGGGGGGCGGGCGGGTGGGTTGGCT CTTCATGCCTGTGCCC
apHAT610	GTTGCTCGTATTTAGGGCGGGGGGGCGGGGGAGGAGGTGGCGGGAATA TCCATTGTCTACACCAGTCTTCATCCGC
apHAT610T	GGGCGGGGGGGCGGGGGAGGAGGTGGCGGGAATATCCATTGTCTACA CCAGTCTTCATCCGC
20AT	GCGGATGAAGACTGGTGTAAAAAAAAAAAAAAAAAAAATTTTTTTTTTTTTT TTTTTTGCCCTAAATACGAGCAAC

In this paper, western blots have been analyzed using a ChemiDoc Imaging System

Figure 4C

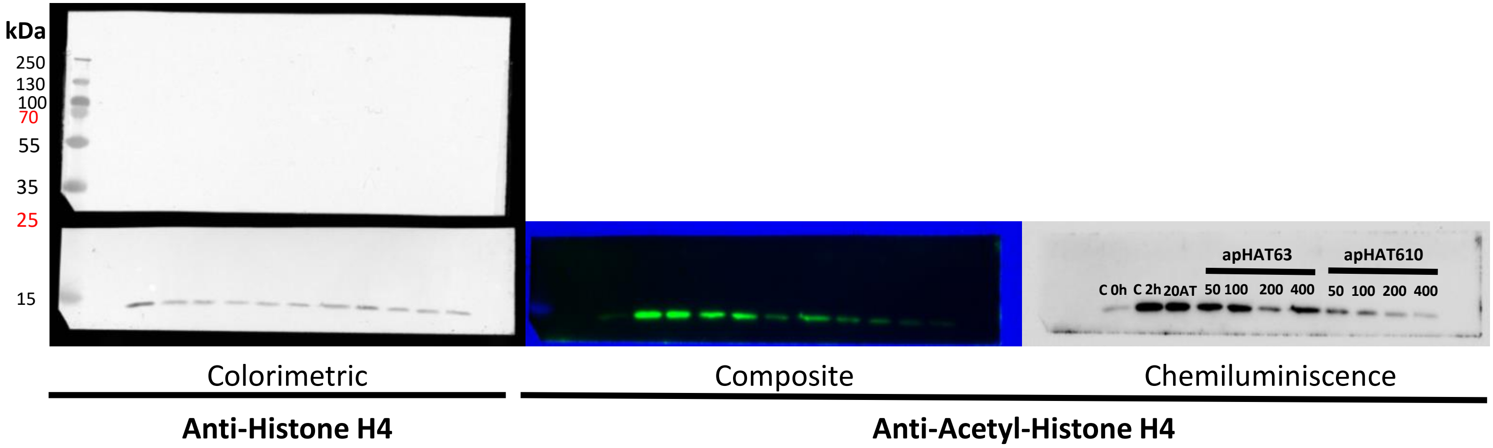

LINE	T0h C	T2h C	20AT	apHAT63 50 nM	apHAT63 100 nM	apHAT63 200 nM	apHAT63 400 nM	apHAT610 50 nM	apHAT610 100 nM	apHAT610 200 nM	apHAT610 400 nM
Anti-Acetyl-Histone H4. (A.U.)	175890	1991862	1654644	1258408	1186566	335410	904545	348466	249492	152524	86802
Anti-Histone H4. (A.U.)	80960022	32953635	30407091	22167145	25143261	25673219	42034354	44998096	27637467	29160417	31518630

Figure 4C

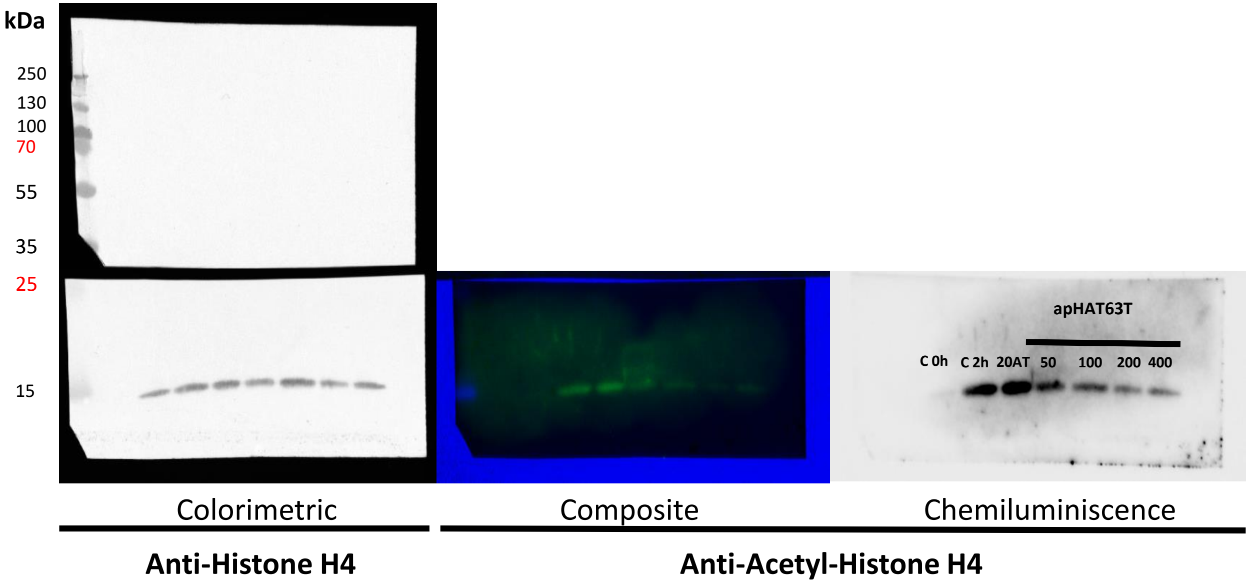

LINE	T0h C	T2h C	20AT	apHAT63T 50 nM	apHAT63T 100 nM	apHAT63T 200 nM	apHAT63T 400 nM
Anti-Acetyl-Histone H4. (A.U.)	207864	2215780	2728600	1434160	1083320	650265	688450
Anti-Histone H4. (A.U.)	50456499	78230607	72812744	59470164	97328088	51192269	58037920

Figure 7

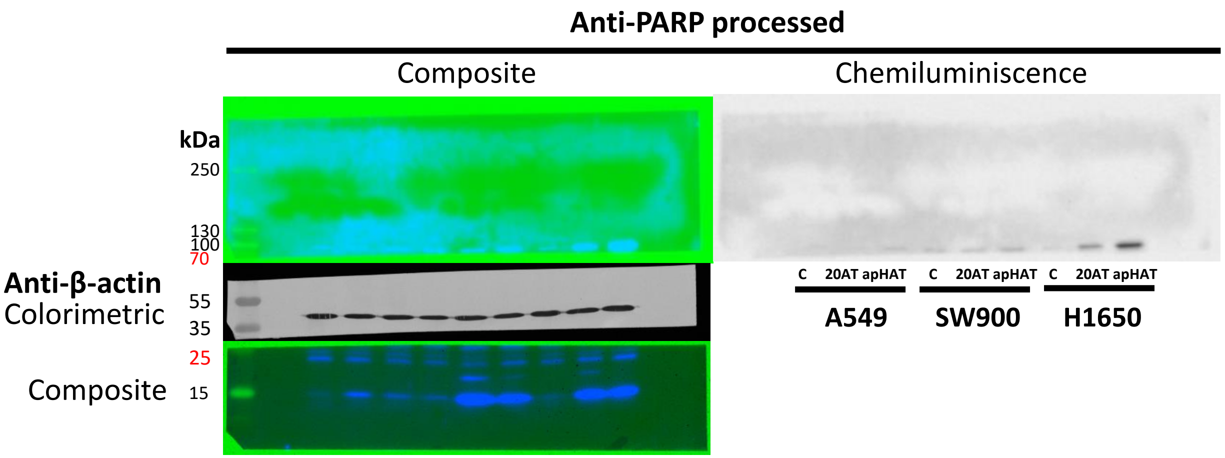

LINE	A549 C	A549 20AT	A549 apHAT610	SW900 C	SW900 20AT	SW900 apHAT610	H1650 C	H1650 20AT	H1650 apHAT610
Anti-PARP processed (A.U.)	486780	607770	628140	549810	384480	536280	357480	607140	1273650
Anti-β-actin (A.U.)	54309840	48908240	49402485	41762288	51143008	49565400	56791254	58725480	71502840

Figure 8A

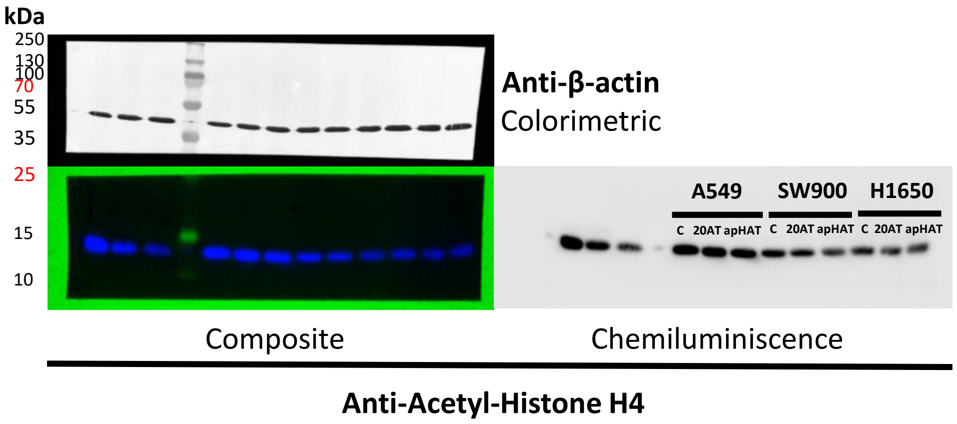

LINE	A549 C	A549 20AT	A549 apHAT	SW900 C	SW900 20AT	SW900 apHAT	H1650 C	H1650 20AT	H1650 apHAT
Anti-Acetyl-Histone H4 (A.U.)	1466528	1287804	1172352	807998	671076	541675	668740	512325	569604
Anti-β-actin (A.U.)	55596326	60374061	64029215	63329069	63666435	68454549	80940387	76912852	78838980

Figure 8B

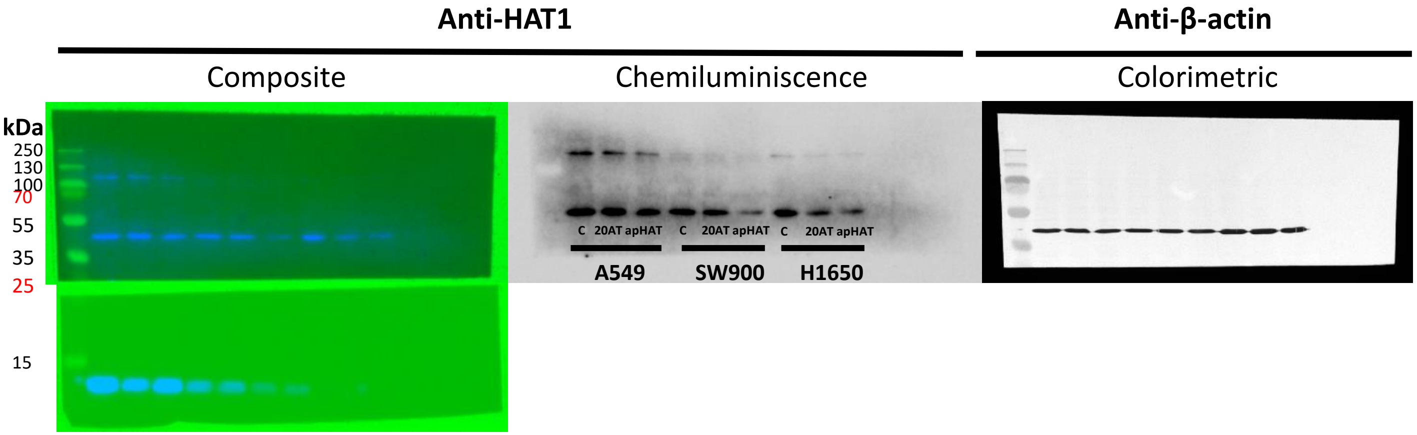

LINE	A549 C	A549 20AT	A549 apHAT	SW900 C	SW900 20AT	SW900 apHAT	H1650 C	H1650 20AT	H1650 apHAT
Anti-HAT1 (A.U.)	215384	200300	155125	169248	135631	55335	186501	80430	65436
Anti-β-actin (A.U.)	36818886	35437388	32057704	34741417	34322532	32370400	42229068	46146096	43470405

Figure S5

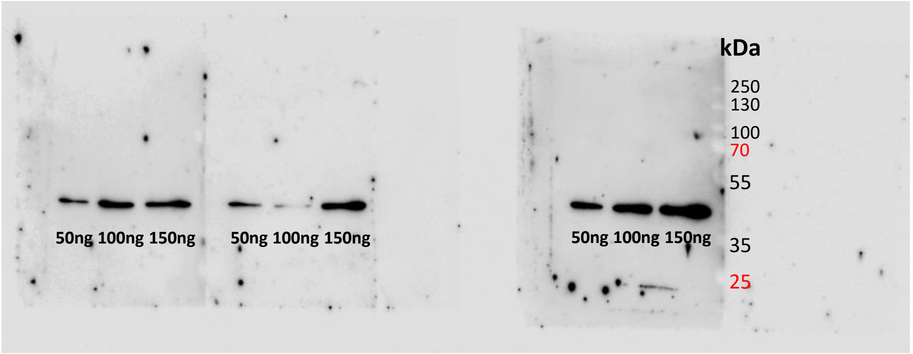

apHAT63 apHAT63T 20AT apHAT610 Control -

LINE	apHAT63 50ng	apHAT63 100ng	apHAT63 150ng	apHAT63T 50ng	apHAT63T 100ng	apHAT63T 150ng	apHAT610 50ng	apHAT610 100ng	apHAT610 150ng
QUANT (A.U.)	956286	2868160	2893380	983892	265860	5365144	2770803	6707434	16566466
